# Cognitive assessment of Brazilian patients with multiple sclerosis: weighing the impact of disability and depressive symptoms

**DOI:** 10.1590/1980-5764-DN-2021-0050

**Published:** 2022-05-23

**Authors:** Patricia Semionato Andrade, Ana Cláudia Rodrigues de Cerqueira, Ana Carolina Colodetti, Felipe da Rocha Schmidt, José Maurício Godoy Barreiros, Antônio Lúcio Teixeira, Leonardo Cruz de Souza

**Affiliations:** 1 Universidade Federal de Minas Gerais, Programa de Pós-Graduação em Neurociências, Belo Horizonte MG, Brazil.; 2 Universidade do Estado do Rio de Janeiro Ambulatório de Neuroimunologia, Hospital Universitário Pedro Ernesto, Rio de Janeiro RJ, Brazil.; 3 Faculdade Santa Casa Belo Horizonte, Programa de Pós-Graduação, Belo Horizonte MG, Brazil.

**Keywords:** Multiple Sclerosis, Neuropsychological Tests, Cognition, Depression, Memory, Esclerose Múltipla, Testes Neuropsicológicos, Cognição, Depressão, Memória

## Abstract

Multiple sclerosis (MS) is the most common demyelinating disease of the central nervous system. Cognition is not routinely assessed in patients with MS though they frequently have cognitive complaints or dysfunction.

**Objective:**

The aim of this study was to compare the cognitive status of patients with MS with age, sex, and schooling matched controls and to evaluate the potential influence of clinical parameters on cognition.

**Methods:**

A total of 35 patients with MS (mean±SD age 37.9 years±11.44, M/F: 12/23) and 33 healthy controls (mean±SD age 38.8 years±12.6, M/F: 12/21) were enrolled in this study. All subjects underwent a structured clinical assessment and the cognitive tools are as follows: Paced Auditory Serial Addition Test (PASAT), Symbol Digit Modalities Test (SDMT), Rey Auditory Verbal Learning Test (RAVLT), Digit Span, and Verbal Fluency Tests (letters F, A, and S and animal category). Psychopathology was assessed with the Mini International Neuropsychiatric Interview and the Beck Depression Inventory (BDI). The Expanded Disability Status Scale (EDSS) was used for patients.

**Results:**

Patients performed worse than controls in almost all tests, with approximately 70% of patients presenting cognitive impairment. The most affected cognitive domain was episodic memory (45.7%), followed by verbal fluency (42.8%) and information processing speed (22.8%). SDMT was inversely correlated with disease severity, as assessed by the EDSS. Depression did not influence cognitive performance in this cohort.

**Conclusions:**

Cognitive dysfunction is common among patients with MS. While motor impairment was associated with information processing speed, depression did not influence cognitive performance.

## RESUMO

A esclerose múltipla (EM) é a doença desmielinizante mais comum do sistema nervoso central. A cognição não é rotineiramente avaliada nos pacientes apesar da ocorrência frequente de queixas ou disfunção cognitivas.

**Objetivo:**

Comparar o perfil de pacientes com EM com controles pareados por idade, sexo e escolaridade e investigar a potencial influência de parâmetros clínicos na cognição.

**Métodos:**

Trinta e cinco pacientes com EM (idade média±desvio padrão [DP] 37,9 anos±11,44, H/M: 12/23) e 33 controles saudáveis (idade média±DP 38,8 anos±12,6, H/M: 12/21) foram incluídos neste estudo. Todos os participantes passaram por avaliação clínica estruturada e por testagem cognitiva com os seguintes instrumentos: Paced Auditory Serial Addition Test (PASAT), Symbol Digit Modalities Test (SDMT), Rey Auditory Verbal Learning Test (RAVLT), Digit Span e testes de fluências verbais (letras F, A e S e categoria-animais). A psicopatologia foi investigada com a Mini International Neuropsychiatric Interview e com o Beck Depression Inventory (BDI). A Expanded Disability Status Scale (EDSS) foi aplicada nos pacientes.

**Resultados:**

Pacientes tiveram desempenho pior que os controles na maioria dos testes — 70% deles tiveram déficit cognitivo. A função cognitiva mais frequentemente afetada foi memória episódica (45,7%), seguida por fluência verbal (42,8%) e velocidade de processamento (22,8%). A pontuação no SDMT correlacionou-se inversamente com a gravidade da doença, medida pela EDSS. A depressão não influenciou o desempenho cognitivo nesta série de pacientes.

**Conclusões:**

Declínio cognitivo é comum em pacientes com EM. Enquanto o déficit motor se associou com a velocidade de processamento, a depressão não influenciou o desempenho cognitivo.

## INTRODUCTION

Multiple sclerosis (MS) is a chronic autoimmune disease of the central nervous system caused by a complex interaction among genetic and environmental factors. Several pathophysiological mechanisms, including neuroinflammation, demyelination, and axonal degeneration, are implicated^1–3^. MS affects approximately 2.5 million people worldwide^2,3^. It is the most common cause of nontraumatic neurological disability in young adults^1,4^.

Cognitive impairment affects a range of 40–70% of patients^2,5–7^. It can be identified in all stages of the disease, including very early in its course, i.e., clinically isolated syndrome^6,8,9^. Typical cognitive impairment involves attention, memory, and executive functions, notably information processing speed (IPS)^5,6,10–12^. Cognitive impairment has a major effect on patients with MS, affecting their daily living and socio-occupational functioning^6,9,12^. Besides contributing to patients’ poor perception of their quality of life, cognitive impairment also reduces adherence to treatment and rehabilitation^10,13,14^. Despite those facts, cognitive function is not routinely assessed in patients with MS. Accordingly, cognitive impairment is frequently overlooked and underdiagnosed in patients with MS^6,10,13,15,16^.

There is an emerging interest on the study of cognitive dysfunction in Brazilian patients with MS^17–20^. For instance, Schmidt et al. showed that patients with less than 3 years of MS diagnosis and low disability have preserved cognitive performance in neuropsychological tests (Rey Auditory Learning Test, Controlled Oral Word Association Test, Hooper Visual Organization Test, and Symbol Digit Modalities Test (SDMT)), except for the number of errors in the SDMT^21^. Conversely, Damasceno et al. showed that patients with relapsing-remitting MS evolved with cognitive decline even if they had minimal or no evidence of disease activity^22,23^.

Understanding the profile of MS cognitive dysfunction in Brazilian patients is important given the particularities of this population, including its unique genetic background and limited formal literacy. Therefore, the aims of this study were twofold: (i) to compare the cognitive status of MS patients with age, sex, and schooling matched controls and (ii) to evaluate the potential influence of sociodemographic and clinical parameters (focus on depression) on the cognitive performance of these patients.

## METHODS

### Subjects

We enrolled 35 patients with relapsing-remitting MS diagnosis according to revised 2010 McDonald criteria^24^. These patients were followed at the Neuroimmunology Outpatient Clinic, Pedro Ernesto University Hospital (HUPE), UERJ, Rio de Janeiro, Brazil. For comparison, 33 healthy subjects who matched by sex, age, and educational level were also invited to participate in the study.

All individuals aged 18–65 years and had at least 5 years of formal education. We did not include subjects who had any preexisting condition (e.g., intellectual disability and dementia) that could potentially interfere with neuropsychological assessment. The exclusion criteria were the Expanded Disability Status Scale (EDSS) value of >7.5 or any relapse or steroid therapy within 2 months before the clinical assessment. Volunteers were not included if they had a clinical diagnosis of major depression, as assessed by the Mini International Neuropsychiatric Interview^25^, or had a score ≥25 in the Memory Complaint Questionnaire^26^ or failed on the Mini-Mental State Examination^27^ or on the component A7 of Rey Auditory Verbal Learning Test (RALVT)^28,29^.

This study was approved by the HUPE Ethics Committee, and all participants gave written informed consent.

### Clinical and cognitive evaluation

We collected sociodemographic and clinical data from all subjects. Neuropsychological tests were selected based on the most impaired cognitive domains in patients with MS^5,6,10^. Paced Auditory Serial Addition Test (PASAT), 3 seconds version^30^, was chosen to assess sustained attention, working memory, and IPS; Symbol Digit Modalities Test (SDMT)^31^ to assess sustained attention and IPS; Rey Auditory Verbal Learning Test (RAVLT)^28,29^ to evaluate learning and verbal episodic memory; Digit Span Test^32^ to assess attention and working memory; and F-A-S and animal category^33,34^ to assess verbal fluency.

Test scores below 1.5 SD of the Brazilian population normative data mean were considered altered. Cognitive impairment was defined as a failure in at least one of the following tests: Component A7 of RAVLT, PASAT, and SDMT^35,36^.

### Data analysis

Database and statistical analyses were performed with Statistical Package for the Social Sciences (SPSS) software, version 19.0. The level of significance adopted was 5%. Group comparisons on clinical and cognitive tests were performed with Mann-Whitney and χ^2^ tests. Correlation between variables was analyzed by the Spearman test.

## RESULTS

A total of 52 patients were originally invited to participate in the study, but only 35 met inclusion/exclusion criteria. Table 1 shows the sample’s sociodemographic and clinical profile. MS group was predominantly composed of young women who developed the first symptoms around 30 years old. They had less than 10 years of disease and mild neurological impairment. The most frequent treatment for MS was interferon-beta. Approximately 30% had major depression, characterized mainly by mild symptoms.

**Table 1:** Sociodemographic and clinical profile of controls and patients.

			Controls (n=33)	Patients (n=35)	p-value
	Age (years)	Mean±SD	38.82±12.6	37.91±11.44	0.94*
		(min–max)	(21–63)	(18–61)	
		Median [IQR]	36 [27.5–50.5]	33 [28–49]	
	Education (years)	Mean±SD	12.7±2.7	11.6±2.4	0.06*
		(min–max)	(5–15)	(5–15)	
		Median [IQR]	14 [11–15]	11 [11–12]	
Sex (%)		Female	63.6%	65.7	0.85**
	EDSS	Mean±SD	NA	2.7±1.9	NA
		(min–max)		(0–6.5)	
		Median [IQR]		2.5 [1–4.5]	
	MS first symptoms (years)	Mean±SD	NA	29.7±10.3	NA
		(min–max)		(13–60)	
		Median [IQR]		27 [24–37]	
	Disease duration (years)	Mean±SD	NA	8.2±5.3	NA
		(min–max)		(1–22)	
		Median [IQR]		8 [3–12]	
	Medication (%)	Interferon-β	NA	57.1	NA
		Glatiramer		22.9	
		Fingolimode		2.9	
		Natalizumab		2.9	
		Azathioprine		2.9	
		No medication		11.4	
Depression (MINI) (%)		Depression	0	28.6	**0.001** **
	BDI	Mean±SD	8±5.6	11.8±9.2	0.10*
		(min–max)	(0–28)	(0–39)	
		Median [IQR]	7 [6–11]	10 [6–16]	

SD: standard deviation; IQR: interquartile range;

* Mann-Whitney;

** χ^2^; EDSS: Expanded Disability Status Scale; BDI: Beck Depression Inventory; MINI: Mini International Neuropsychiatric Interview; min: minimum value; max: maximum value; n: sample size; NA: not applicable; p: level of confidence. Significant values (p<0.05) in bold.

Patients had worse cognitive performance in almost all neuropsychological tests when compared with healthy controls, except for the Digit Span Test, both versions, and variables A1 and B1 of RALVT (Table 2). The most significant results were related to the following tests: PASAT, SDMT, Verbal Fluency Test (letter F, FAS total, and animal category), and RALVT (A2–A6, total A1–A5 and late recall).

**Table 2:** Results of the neuropsychological tests between groups.

Test		Controls (n=33)	Patients (n=35)	p-value
PASAT	Mean±SD	39.6±13.3	31.4±12.1	**0.008**
	(min–max)	(11–60)	(6–54)	
	Median [IQR]	41 [29.5–49.5]	30 [21–41]	
SDMT	Mean±SD	56.3±13.5	45±16	0.003
	(min–max)	(17–80)	(17–73)	
	Median [IQR]	55 [46–67]	43 [33–57]	
Direct SPAN	Mean±SD	8.1±2.7	8.2±2.3	0.69
	(min–max)	(4–14)	(4–14)	
	Median [IQR]	7 [6–10]	8 [6–10]	
Inverse SPAN	Mean±SD	5.4±2.9	5.1±2.1	**0.30**
	(min–max)	(3–11)	(0–9)	
	Median [IQR]	4 [4–5]	5 [4–6]	
Fluency (F)	Mean±SD	14.9±4.2	11.9±5	**0.007**
	(min–max)	(5–26)	(4–24)	
	Median [IQR]	15 [12.5–18]	11 [7–16]	
Fluency (A)	Mean±SD	13.1±4.2	10.7±4.5	**0.015**
	(min–max)	(3–22)	(3–21)	
	Median [IQR]	14 [10.5–16]	10 [8–14]	
Fluency (S)	Mean±SD	12.8±4.2	10.6±4.1	**0.03**
	(min–max)	(3–19)	(3–19)	
	Median [IQR]	13 [10–16]	11 [7–14]	
Total FAS	Mean±SD	40.8±11.1	33.2±11.6	**0.008**
	(min–max)	(13–63)	(15–61)	
	Median [IQR]	41 [35.5–48]	33 [24–42]	
Animals fluency	Mean±SD	21.9±5.9	17±4.3	**0.001**
	(min–max)	(12–35)	(9–28)	
	Median [IQR]	21 [17.5–26.5]	17 [14–20]	
RALVT – A1	Mean±SD	6.2±1.9	5.4±1.5	0.72
	(min–max)	(1–10)	(2–8)	
	Median [IQR]	6 [5–7.5]	6 [4–7]	
RALVT – A2	Mean±SD	9.7±2	7.9±1.9	**0.001**
	(min–max)	(5–13)	(4–12)	
	Median [IQR]	10 [8–11]	8 [7–9]	
RALVT – A3	Mean±SD	11.2±1.9	9.4±2.2	**0.003**
	(min–max)	(8–14)	(4–13)	
	Median [IQR]	11 [10–13]	10 [8–11]	
RALVT – A4	Mean±SD	12.2±1.6	10.1±2.3	**0.000**
	(min–max)	(9–15)	(6–15)	
	Median [IQR]	12 [11–13.5]	10 [9–12]	
RALVT – A5	Mean±SD	12.7±1.8	11±2.5	**0.004**
	(min–max)	(10–15)	(6–15)	
	Median [IQR]	13 [11–14.5]	11 [9–13]	
Total A1–A5	Mean±SD	52.2±7.4	44±9.1	**0.000**
	(min–max)	(40–65)	(25–62)	
	Median [IQR]	53 [45–59]	43 [39–49]	
RALVT – B1 (interference)	Mean±SD	5.9±2.2	5.1±1.6	0.20
	(min–max)	(3–12)	(1–8)	
	Median [IQR]	6 [4–7]	5 [4–6]	
RALVT – A6	Mean±SD	10.6±2.5	8.4±3.4	**0.007**
	(min–max)	(6–15)	(1–15)	
	Median [IQR]	10 [8–12.5]	8 [7–11]	
RALVT – A7 (late recall)	Mean±SD	10.8±2.7	7.6±3.4	**0.000**
	(min–max)	(6–15)	(1–14)	
	Median [IQR]	11 [9–13]	8 [5–10]	
RALVT (recognition)	Mean±SD	28.7±1.5	28.3±4	**0.018**
	(min–max)	(24–30)	(7–30)	
	Median [IQR]	29 [28.5–30]	28 [27–30]	

SD: standard deviation; IQR: interquartile range; PASAT: Paced Auditory Serial Addition Test; SDMT: Symbol Digit Modalities Test; RALVT: Rey Auditory Verbal Learning Test; min: minimum value; max: maximum value; n: sample size; p: level of confidence; Significant values (p<0.05) in bold.

Cognitive impairment was detected in 68.6% of patients. Episodic memory (late recall) was affected in 45.7% of patients, information processing speed in 22.8% of patients, and verbal fluency in 42.8% of patients. Patients with or without cognitive impairment did not differ in sociodemographic and clinical parameters (Table 3).

**Table 3:** Comparisons between patients with and without cognitive impairment.

		Preserved cognition (n=11, 31.4%)	Impaired cognition (n=24, 68.6%)	p-value
Age (years)	Mean±SD	36±11	38.7±11.7	0.70*
	Median [IQR]	33 [28–47]	34.5 [28–49.7]	
Education (years)	Mean±SD	11.4±1.2	11.6±2.8	0.40*
	Median [IQR]	11 [11–12]	11.5 [11–15]	
EDSS	Mean±SD	2.9±2	2.7±1.9	0.84*
	Median [IQR]	2.5 [1–5]	2.2 [1–3.8]	
Disease duration (years)	Mean±SD	7.,1±4.9	8.6±5.4	0.50*
	Median [IQR]	6 [2–13]	8.5 [3.2–11.7]	
Depression (MINI) (%)	n – %	2 – 18%	8 – 33%	0.35**
BDI	Mean±SD	12.2±8.8	11.6±9.8	0.64*
	Median [IQR]	10 [6–15]	9 [4–16.7]	
Sex (%)	Female	8–35%	15–65%	0.55**
	Male	3–25%	9–75%	
Medication (%)	Interferon-β	46%	63%	0.51**
	Glatiramer	27%	21%	
	Fingolimode	9%	0%	
	Natalizumab	0%	4%	
	Azathioprine	0%	4%	
	No medication	18%	8%	

SD: standard deviation; IQR: interquartile range;

* Mann-Whitney;

** χ^2^; EDSS: Expanded Disability Status Scale; MINI: Mini International Neuropsychiatric Interview; BDI: Beck Depression Inventory; n: sample size; p: level of confidence.

In an exploratory analysis, neuropsychological performance was correlated with clinical parameters. The only significant association that emerged was the inverse correlation between EDSS and SDMT (rho=-0.58; p=0.003).

## DISCUSSION

Cognitive dysfunction has been recognized as a central problem in patients with MS^2,5,6,12^ By affecting daily living activities, work capacity, and social relationships, it h.as a significant effect on the patients’ quality of life^9,12,14^. Its early recognition and management may have a relevant meaning for patients with MS^9,37^.

In the current study, patients with MS performed worse compared to controls in almost all neuropsychological tests. The most significant differences were in the PASAT, SDMT, Verbal Fluency Test (total FAS and animal category), and RALVT (learning and late recall), corroborating the concept that IPS and episodic memory/learning are the most affected cognitive domains in patients with MS^5,6,10–12^. While 45.7% of the patients with MS had deficits in episodic memory, IPS was impaired in 22.8% of patients who performed below the normative data for the PASAT (17.1%) and/or SDMT (11.4%). Similar results have been described in other Brazilian and Latin American studies: 22.5% in SDMT according to the study by Negreiros et al.^38^; 12 and 21.8%, respectively, in PASAT and SDMT, according to the study by Caceres et al.^39^ When analyzing verbal fluency, 42.8% of impairment was observed. Negreiros et al.^38^ found a similar rate of 40.7%. In contrast, other studies reported lower indexes of around 16–19%^36,39,40^.

The performance in the Digit Span was similar between patients and controls. A similar finding was reported by Balsimelli et al.^41^, while Negreiros et al.^38^ reported differences in both direct and inverse component scores of the Digit Span. In international studies on patients with MS, the direct component is usually not impaired, whereas deficits have been reported in the indirect component^5,32,42^. Differences in sample characteristics (e.g., disease severity and comorbidities) might explain these discrepant results.

Cognitive impairment was identified in approximately 70% of patients. Although this frequency is high, it is in accordance with data from international^5,6^ and national studies^38,43^. It is worth emphasizing that interpretation and comparison of the studies on cognitive impairment in MS are challenging as there is no consensus about its definition. Some authors define cognitive dysfunction as the impairment in one of the three tests, regardless of the cognitive domain, in a series of tests applied^12,44^. Neuropsychological tests also vary significantly among studies. Another issue is the definition of neuropsychological test alteration. In general, an altered result is determined by the performance below a threshold chosen by authors (SD below the normative population or control data mean). This choice has varied among Brazilian studies: from 1 SD^45,46^, to 1.5 SD^40,43^, up to 2 SD^36,47^ below the mean.

Approximately 30% of patients had a clinical diagnosis of depression, a figure similar to previous studies^39,45,47^. We did not observe an influence of depression on the cognitive performance of patients with MS. In addition, there was no significant correlation between BDI and neuropsychological scores, consistent with some studies^45^. In contrast, some authors have shown a significant, but weak, association between depression and cognitive performance in patients with MS^40,48^.

In the current sample, as cognitive dysfunction in patients with MS does not seem to be influenced by mood symptoms, we hypothesized that it is primarily a consequence of MS-induced changes in the brain structure and/or functioning. Corroborating in part this assumption, we found an inverse correlation between SDMT and EDSS, an index of disease-related disability. The degree of physical disability can inform about the extension and/or severity of brain damage and, as a consequence, cognitive functioning. Physical disabilities have been associated with cognitive performance in several Brazilian and international studies^35,40,43,45^, but not in all studies^36,38,47^.

Disease duration did not influence cognitive performance in our sample, in contrast to some of the Brazilian studies^36,45^. Actually, some studies^35,40^ have reported an inverse correlation between disease duration and cognitive scores, especially SDMT. The exclusion of patients with EDSS>7.5, who usually have a longer disease duration, might explain this contradicting result.

While our study has strengths, such as well-established inclusion and exclusion criteria ruling out clinically defined depression and cognitive impairment, it also has clear limitations. First, our study involved a relatively small (n=35) sample of patients lacking neuroimaging results coupled with clinical assessment. Moreover, it was not possible to control for different immunomodulatory treatments. Most patients were treated using interferon and glatiramer (Table 1), which increases the chance of subtle MS activity detected only through sensitive neuroimaging techniques that can negatively affect cognitive performance^49^. While our sample size is comparable to former Brazilian studies^35,43,46,47^, it is more homogeneous, highlighting the presence of cognitive impairment even in patients with higher formal education and without clinically defined depression, a condition that influences cognitive performance. Another limitation concerns the cognitive domains tested. We assessed only domains that are classically implicated in patients with MS, such as attention, episodic memory, IPS, and executive function, using well-studied tools in this context. The expansion of neuropsychological testing to other functions, such as visuospatial skills and social cognition, and the use of new tools would provide a more comprehensive understanding of MS-related cognitive profile.

In conclusion, our results support previous findings, showing that cognitive impairment is common among patients with MS without clinically defined depression, suggesting an important role played by MS pathology in cognition.
